# Moderate Load Eccentric Exercise; A Distinct Novel Training Modality

**DOI:** 10.3389/fphys.2016.00483

**Published:** 2016-11-16

**Authors:** Hans Hoppeler

**Affiliations:** Department of Anatomy, University of BernBern, Switzerland

**Keywords:** eccentric exercise, rehabi, sarcopenia, COPD, alpine skiing

## Abstract

Over the last 20 years a number of studies have been published using progressive eccentric exercise protocols on motorized ergometers or similar devices that allow for controlled application of eccentric loads. Exercise protocols ramp eccentric loads over an initial 3 weeks period in order to prevent muscle damage and delayed onset muscle soreness. Final training loads reach 400–500 W in rehabilitative settings and over 1200 W in elite athletes. Training is typically carried out three times per week for durations of 20–30 min. This type of training has been characterizes as moderate load eccentric exercise. It has also been denoted RENEW (Resistance Exercise via Negative Eccentric Work by LaStayo et al., 2014). It is distinct from plyometric exercises (i.e., drop jumps) that impose muscle loads of several thousand Watts on muscles and tendons. It is also distinct from eccentric overload training whereby loads in a conventional strength training setting are increased in the eccentric phase of the movement to match concentric loads. Moderate load eccentric exercise (or RENEW) has been shown to be similarly effective as conventional strength training in increasing muscle strength and muscle volume. However, as carried out at higher angular velocities of joint movement, it reduces joint loads. A hallmark of moderate load eccentric exercise is the fact that the energy requirements are typically 4-fold smaller than in concentric exercise of the same load. This makes moderate load eccentric exercise training the tool of choice in medical conditions with limitations in muscle energy supply. The use and effectiveness of moderate load eccentric exercise has been demonstrated mostly in small scale studies for cardiorespiratory conditions, sarcopenia of old age, cancer, diabetes type 2, and neurological conditions. It has also been used effectively in the prevention and rehabilitation of injuries of the locomotor system in particular the rehabilitation after anterior cruciate ligament surgery.

## Background

There are generally acknowledged modalities to train either aerobic capacity (endurance training) or the capacity of muscles to develop force (strength training). Endurance training aims at improving the overall capacity of the organism to perform aerobic work by activities such as running, bicycling, or cross-country skiing involving a significant muscle mass. Effective exercise intensities are characterized by individual target heart rates to be achieved in training sessions lasting typically 30 min or more for 5 days per week. Strength training targets individual muscles or muscle groups whereby effective protocols use 1–3 sets of 8–12 near maximal contractions per individual muscle of muscle groups in 2–3 training sessions per week (Garber et al., 2011). Endurance and strength training are thus opposite ends of a spectrum of training modalities with the accent either on the metabolic activity of the entire organism (endurance) or the maximal performance capacity of individual muscles. Endurance training can thus loosely be described as “low load—high repetitive” training with a weekly performance of thousands of muscle contractions while “high load—low repetitive” strength training protocols is done with only a few hundred very hard contractions per week. The basic “endurance” and “strength” training modalities appear in uncountable guises whereby the individual load, the frequency of the training sessions and there duration or repetition is varied according to specific training purposes or according to believes of training experts.

Over the last 10 years an additional training modality, HIT training (high-intensity interval training), has become popular as a time-efficient mean for improving cardiorespiratory and metabolic capacities (Buchheit and Laursen, 2013). In HIT training repeated bouts of 15 s to 3 min of maximal or near maximal work intensities are carried out several times weekly. HIT-training has been promoted either as a sole mean of exercise training or as an adjunct to more classical low—load expansive types of endurance training. HIT training has been shown to be as effective as more classical endurance training programs in improving VO2max (Milanović et al., 2015).

The purpose of the current review is to describe and add a fourth distinct training modality to the three accepted generic exercise training modalities presented above: *Moderate Load Eccentric Exercise*. The review is aimed at exercise scientists, health professionals, trainers and athletes to raise awareness of a novel training paradigm, outlining physiological properties, and potential fields of application. This is done in view of an apparent lack of general appreciation of the role of eccentric muscle activity in human locomotion, training and rehabilitation.

In moderate load eccentric training the emphasis is completely on eccentric muscle activity see Hoppeler (2014). In eccentric contractions, at any given level of effort, the load that is applied to a muscle is larger than the torque that it produces. Under these conditions the activated muscle undergoes lengthening. This muscle behavior is called active muscle lengthening or *eccentric contraction*. In eccentric contractions, a muscle performs negative work i.e., it decelerates an object or it absorbs potential energy such as when lowering a load or when walking downhill.

**Figure 1:**
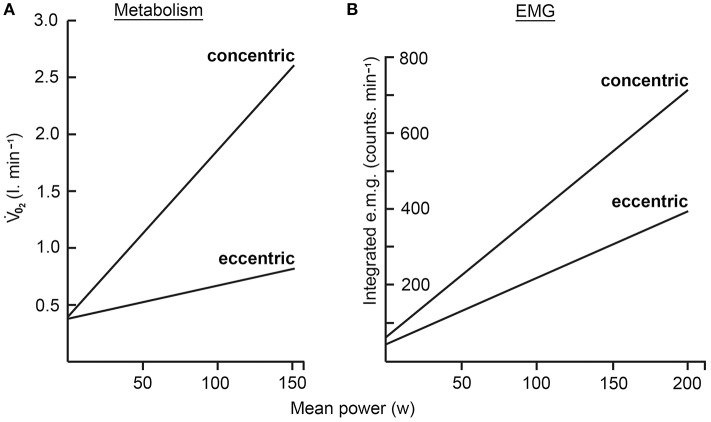
**(A)** Indicates with the difference in slope that energy demand as expressed by oxygen consumption is much lower in eccentric than in concentric contractions. **(B)** Demonstrates that eccentric contractions need much lower central nervous activation expressed as EMG activity to produce similar torques than concentric contractions (with permission from Hoppeler, 2014).

The physiological properties differ vastly between concentric and eccentric contractions as discussed below. An important aspect of eccentric muscle work is its reduced energetic requirement (Figure 1). Typically, there is an approximately 4-fold lower energy requirement when walking downhill than when walking uphill over the same gradient. Eccentric exercise thus presents a modality by which high loads on muscle tissue can be combined with small energy requirements for muscle contraction. It is generally accepted that (high) mechanical load is of paramount importance for muscle maintenance and adaptation (Hoppeler, 2016). Thus, eccentric training protocols are beginning to be explored for patient situations in which central (cardiorespiratory) limitations constrain muscle work such as in COPD (Chronic Obstructive Pulmonary Disease), heart insufficiency, cachexia, or sarcopenia.

## Specifics of eccentric exercise

**Figure 2:**
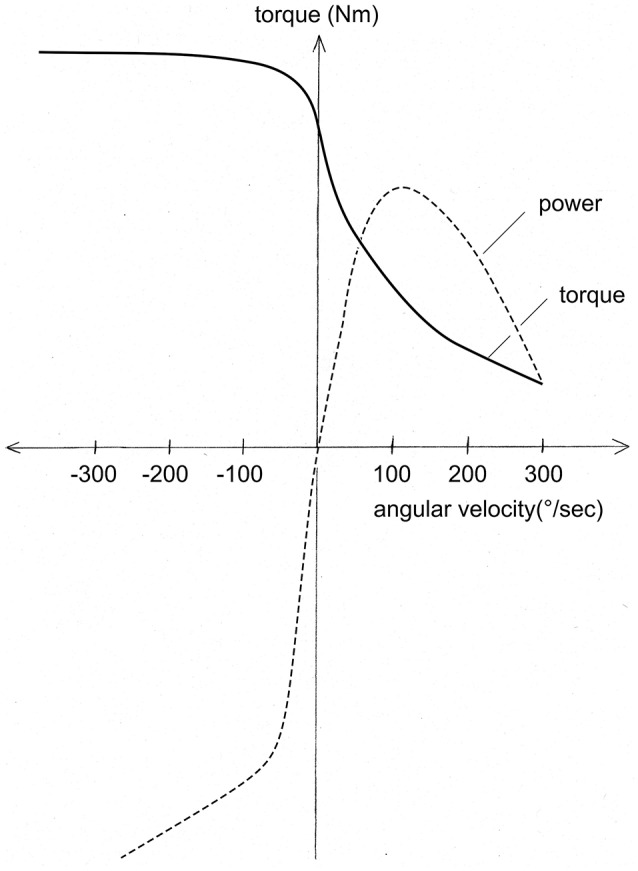
**As indicated by the continuous line, torque decreases with increasing (positive) angular velocity while it increases and then stays constant with negative angular velocities**. As a consequence, power (angular velocity × torque) has an optimum in concentric contractions and the decreases at higher angular velocities. Negative power increases first and then continues to increase with higher (negative) angular velocities. This indicates, that extremely high values of negative power can be achieved putting muscle tissue at risk (with permission from Hoppeler, 2014).

A fundamental and consequential aspect of eccentric contractions consists in the difference of the force-velocity relationship for positive and negative shortening velocities (Figure 2). While torque decreases hyperbolically with increasing shortening velocities of contraction (Hill, 1938), in eccentric contractions, torque increases initially, and then stays constant and high for increasing lengthening velocities. As a consequence, during rapid lengthening a muscle is able to absorb several-fold more torque than what it generates when shortening at the same velocity. Power generated during eccentric contractions increases linearly with increasing lengthening velocity while in concentric contractions it decreases hyperbolically with higher shortening velocities. The extremely high torque that can be generated during rapid eccentric contractions puts muscles and tendons at risk. Single massive eccentric events such as drop jumps, can result in acute structural muscle lesions including tendon ruptures, or even avulsion of muscles (Celli, 2015). Repetitive eccentric events have been shown to be responsible for what is generally referred to as DOMS [delayed onset muscle soreness; see Lewis et al. (2012)]. DOMS is assumed to be the sequel of micro-damage incurred by muscle tissue during repeated low-level eccentric contractions for instance during long-term downhill walking. DOMS is often associated with the release of muscle proteins such as creatine kinase or myoglobin into the blood stream as well as with prolonged decreases in muscle performance capacity. Muscle damage during eccentric contractions and DOMS can largely be prevented with progressive load eccentric training protocols due to the “repeated bout effect” (McHugh, 2003). Whereby, the physiological mechanism of the repeated bout effect has not yet been established (Hoppeler, 2014).

The reduced energy requirement of eccentric exercise as compared to concentric exercise has been mentioned above. High muscle loads at a low metabolic cost makes continuous moderate load eccentric exercise potentially attractive as a training modality in medical conditions with a centrally limited performance capacity. As indicated by Panel B in Figure 1 a further characteristic of eccentric exercise is a reduction of central nervous activity for similar workloads in eccentric than in concentric exercise as reviewed by Bigland-Ritchie and Woods (1976). Figure 1 indicates that EMG activity for eccentric contractions is roughly half of that for concentric contractions. As a consequence, there is a reduction of fine motor control in eccentric torque production. This has implications for eccentric coordination, making fine control of movements more difficult as fewer motor-units are involved.

Eccentric overload training protocols have also been used to explore muscle architectural changes. Pure concentric and pure eccentric training regimes have both been shown to increase muscle fascicle length as well as pennation angle (Blazevich et al., 2007). Using conventional vs. eccentric-only resistance training (Reeves et al., 2009) found eccentric exercise to favor fascicle length increase. By contrast, conventional strength training increased pennation angle more than eccentric training. Muscle thickness was found to increase similarly with both training modes. In a study of eccentric training of the biceps femoris, a 34% increase in fascicle length was combined with an 5% increase of the range of motion in the knee joint (Potier et al., 2009). It thus appears that both concentric and eccentric training regimes can change architectural features of muscle with high load eccentric exercise having a predilection to increase fascicle length. This is an important consideration when muscles are shortened as seen in some sports or in rehabilitation. Eccentric exercise has thus the potential to shift the optimum of the length-tension relationship to longer muscle length with implications for injury prevention and athletic performance (Brughelli and Cronin, 2007).

Experiments with transcranial magnetic stimulation of the brain report differences in corticospinal excitability with concentric and eccentric contractions. This could indicate different involvement of higher-order cortical centers in eccentric exercise (Sekiguchi et al., 2007). Using submaximal and maximal concentric and eccentric contractions of the biceps brachii at 30°/s, (Fang et al., 2004) found electroencephalogram (EEG)-derived movement-related cortical potentials (MRCP) to occur earlier and to be greater in eccentric contractions. They took this to indicate that eccentric movements need longer time for early preparation and more cortical drive for movement execution. Taken together the literature contains limited evidence that our central nervous system controls eccentric contractions differently from concentric contractions. These differences can be detected on the level of the working muscle as well as on the cortical level. While eccentric contractions need less EMG activation, they seem to need more time for preparation and more activation from centers in the cortex. The observations on the differences in cortical involvement of eccentric contractions when compared to concentric contractions is supported by the every-day observations that most subjects find it difficult to coordinate eccentric bicycling tasks and are unable to coordinate eccentric bicycling at a pace higher than 90 RPM; an easy task when bicycling concentrically. It is unclear why we are unable to learn the pattern of eccentric muscle activations necessary to resist eccentric loading in a coordinated manner at pedal cadences higher than 90 RPM.

## Modes of eccentric exercise

It seems convenient and meaningful to distinguish eccentric contraction events by the time over which they occur. Eccentric contractions lasting for (some hundreds of) milliseconds such as drop jumps and similar plyometric exercises are most prone to massive and acute muscle damage. It can be calculated that a drop jump from 1 m in altitude loads the Achilles tendon and associated muscles with several thousands of watt of negative power to decelerate the body upon landing on the ground. The current evidence suggests that drop jumps and plyometric exercises are effective in increasing kicking distance, speed and jumping ability in soccer players (Bedoya et al., 2015). Due to the inherent risk of muscle damage in plyometric exercises, the literature suggests specific habituation training and knowledgeable supervision (Hess, 2010).

Eccentric contractions are an integral part of all classical strength training protocols. In these protocols near maximal concentric and eccentric contractions lasting approximately 1 s are used to lift and lower weights. As can be seen in Figure 2, even at low angular velocities, muscles have a larger capacity to produce torque during the eccentric phase of the exercise. To equally load muscle during the concentric and the eccentric part of the exercise it is necessary to add some 20–30% of the concentric load during the eccentric phase of the exercise. Strength training with an increased load during the eccentric phase is denoted eccentric “overload” training. Eccentric overload can be achieved by an aide that adds weight after the completion of the concentric phase of muscle contraction. Recently, a number of strength training devices have become available that use various techniques to increase muscle load during the eccentric phase of the exercise motion. The available evidence indicates that eccentric overload exercise can be more effective in promoting strength and muscle mass gains and that this capacity is related to the higher forces that can be achieved during eccentric muscle actions. However, the authors of a meta-analysis (Roig et al., 2009) find a remarkable specificity of eccentric training with regard to the mode of contraction as well as contraction velocity. This can compromise transferability of eccentric strength gains to functionally relevant athletic movements. It has been suggested that some of the observed training specificity is likely of neuronal origin (Vikne et al., 2006). It is currently unclear to what extent apparent strength gains in eccentric exercise are due to changes in muscle structure or changes in the neuronal activation pattern—and how these compare to the changes observed in concentric strength training. Eccentric overload training has further been achieved by so called Yo-Yo devices. These have originally been designed for use in space (Berg and Tesch, 1994). These authors have developed gravity independent machines that allows for concentric/eccentric movements. They use a flywheel that is accelerated during the concentric phase of the exercise and brought to a standstill in a (shorter) eccentric phase, providing eccentric overload. In a similar vein, there are training machines based on a motor assisted barbell that allows for eccentric overload with specific load and velocity profiles (Frohm et al., 2005).

We have used the term “moderate load eccentric exercise” to denote eccentric exercise protocols lasting between 5 and 30 min (Hoppeler, 2014). We see “moderate load eccentric exercise” as a distinct novel training modality with specific properties in terms of muscle tissue and systemic adaptations. The physiological properties, safety considerations and the practical use of moderate load eccentric exercise in sport and rehabilitation are discussed below.

## Initial implementation of moderate load eccentric exercise

Initially, we used moderate load eccentric exercise protocols to investigate the physiological properties of continuous eccentric vs. continuous concentric exercise and the different effects that these distinct training regimes would have on muscle function and structure (Lastayo et al., 1999; LaStayo et al., 2000). The initial exploratory studies were done with the perspective of using a training technique which combined high mechanical loads on muscle tissue with a low cardiovascular effort. They were done on active young subjects of both sexes. In both studies, subjects were randomized to a “concentric” and to an “eccentric” training group. Being aware of the fact that eccentric exercise was prone to induce DOMS we realized that we needed to exploit the “repeated bout” effect to protect subjects from muscle soreness and damage. In preliminary exercise sessions on a calibrated custom built eccentric ergometer we found that as little as 10 min of 100 W eccentric loads could produce noticeable muscle soreness in subjects unaccustomed to eccentric exercise. This was quite surprising as 100 W of eccentric load is easily tolerated taxing the metabolism by the equivalent of a mere 25 W of concentric work. We started the 6–8 weeks progressive training regimes with only 2 × 15 min of training per week at loads close to 100 W. The aim of the studies was to train subjects in the final weeks of the training protocol at a similar metabolic load either as estimated by oxygen consumption (Lastayo et al., 1999) or by heart rate (LaStayo et al., 2000). As a consequence of matching metabolic load in these studies the concentric group trained at close to 100 W (positive) mechanical load while the eccentric group trained at close to 500 W (negative) mechanical load in the final weeks of the training protocol. Due to the progressive character of the eccentric loading protocol, leg pain (DOMS) occurred only during the first 3 weeks of training and was minimal (not more than three on a visual analog scale of 0, no pain, to 10, maximal pain). No pain was noted for subjects working concentrically. Isometric strength of the knee extensors increased significantly in the eccentric group only (Lastayo et al., 1999; LaStayo et al., 2000). The structural analysis of muscle biopsies further indicated a significant increase of the muscle cross-sectional area also in the eccentric group only (LaStayo et al., 2000).

These initial “proof of concept” studies indicated the feasibility of moderate load eccentric exercise training studies. It was demonstrated that at a moderate metabolic load achieved with concentric exercise at 100 W, eccentric exercise could be carried out at approximately 500 W. It was demonstrated that these moderate mechanical loads could be achieved without undue muscle soreness and without damage to the muscle tissue provided an initial progressive ramping protocol was respected. The initial studies also indicated that moderate load eccentric exercise carried out at a metabolic load equivalent to about 100 W was capable of increasing muscle strength, cross-sectional area and fiber size to a similar degree as conventional strength training. This was a surprising finding as these strength and volume increases observed in the knee extensor muscles were achieved without the need for near maximal contractions necessary under classical strength training conditions. In this context it may be noted that classical strength training protocols are carried out at angular velocities between 20 and 60°/s. As a consequence of the low angular velocities and the near maximal forces exerted both during the concentric and the eccentric phase of the movement, joint load in open or closed chain knee extension can be larger than 4500 N (Wilk et al., 1996). In moderate load eccentric exercise, angular velocities are of the order of 240°/s (Rösler et al., 1985) and forces exerted by the knee extensors are not near maximal. This massively reduces joint loads during moderate load eccentric exercise, making this training modality more “joint friendly.”

The overall experience of the studies referred to below indicates that moderate load eccentric exercise is effective in increasing muscle strength and muscle volume when carried out three times per week with initial loads of 50–75 W for initial durations of 5–10 min. Ramping of loads can safely be carried out over a 2–3 weeks period to the desired load which for practical rehabilitation purposes should be between 400 and 600 W. Guidelines for progression protocols for moderate load eccentric exercise in rehabilitative settings are given by LaStayo et al. (2014). We found 20 min to be an adequate duration for an individual training session. As an option in higher load trainings we found four bouts of 5 min to be equally effective and less tiring for subjects (Steiner et al., 2004; Vogt and Hoppeler, 2012).

## Moderate load eccentric exercise and the stiffness of the muscle-tendon unit

In animal and human locomotion, the elastic storage of energy in tendons and muscle is an important mechanism by which organisms can save up to 40% of energy during locomotion (Biewener, 1998). In high speed locomotion energy is stored in the muscle-tendon unit when the foot comes into contact with the ground and extensor muscles perform a stretch-shortening cycle. They release much of the energy stored in the initial stretching phase during the final shortening phase before foot lift off. The stiffness of the relevant muscle-tendon units is tuned to the stride frequency of the individual and largely depends on its mass with smaller animals having higher stride frequencies (Lindstedt et al., 2002). As an individual's mass is not constant over a lifetime it would appear to be of advantage to have a malleable stiffness of the muscle-tendon unit. Furthermore, it has been shown in humans that high running speeds are associated with high leg stiffness as estimated by a hopping jump test (Chelly and Denis, 2001). As athletic exercise training can effectively increase maximum running speed, it could also be hypothesized that leg stiffness could be increased accordingly.

The hypothesis that moderate load eccentric exercise could change the stiffness of the muscle-tendon unit was tested in the initial experiments carried out by LaStayo et al. (2000). In his study the hopping frequency of the subjects performing eccentric exercise was significantly increased by over 10% while the hopping frequency of the subjects training concentrically remained unchanged. Similar results were obtained on high school basketball players subjected to moderate load eccentric exercise training or classical strength training for 6 weeks (Lindstedt et al., 2002). Jumping height remained unchanged in those subjects that performed traditional weight-lifting, while it improved significantly by 8% (+5 cm) in the group that used moderate load eccentric exercise. The contention that moderate load eccentric exercise can increase leg stiffness and thus improve elastic recovery of energy in rebound situation is supported by a study on junior alpine skiers (Gross et al., 2010). The control group performed 1 h weight sessions three times per week while the eccentric group complemented reduced volume weight sessions with 20 min of moderate load eccentric exercise. Lean muscle mass as well as jump height in the counter-movement jump (+6.5%) increased only in the group supplemented with eccentric exercise. Reich et al. (2000) carried out an experiment involving rats undergoing 8 weeks of eccentric exercise by running downhill on a motor driven treadmill at 16 m/min while controls were not exercised. It was found that passive and active lengthening force at all muscle lengths increased significantly in the rats performing eccentric exercise. It was also found that the stiffness of the muscle-tendon unit (in N/mm) was 30% larger in the eccentric group a gain of the capacity to store stretch-energy.

Taken together, a number of small scale animal and human studies indicate the potential of moderate eccentric exercise to increase the active and passive stretch properties of the muscle-tendon unit, leading to an enhanced recovery of elastic strain energy. This results in improved performance in rebound activities such as jumps. However, the mechanism(s) by which this is achieved and the relative contribution of muscles and tendons remains open. A change in titin isoform pattern observed with eccentric exercise in the rat experiment has been taken to suggest a role for this protein in increasing muscle stiffness (Lindstedt et al., 2002).

## Moderate load eccentric exercise in cardiac rehabilitation

Our initial motivation to explore moderate load eccentric exercise was its capability to achieve high loads on muscle tissue at low metabolic costs. The studies under similar metabolic load conditions demonstrated significant functional and structural muscle adaptations at eccentric loads below 500 W. As these significant mechanical loads could be achieved at metabolic loads generally used in cardiac rehabilitation programs (around 100 W) we decided to perform a “proof of principle” study for the use of moderate load eccentric exercise programs in coronary patients. The main initial concern of cardiologists was that eccentric exercise could lead to high pressure in the central circulation.

For this study we choose a group of patients with documented previous myocardial infarction (*n* = 10), and percutaneous transluminal coronary angioplasty (*n* = 9) or coronary bypass surgery (*n* = 3; Meyer et al., 2003). They were prospectively randomized into a concentric and an eccentric training group. Training was carried out at a similar percentage of maximal heart rate (85% of HRmax) in both groups. Training was performed for 8 weeks, three times per week. Ramping of the exercise intensity was done over the first 5 weeks of training. At that time patients working eccentrically worked at 4-fold higher loads than patients on the concentric protocol. After week 5, central hemodynamic variables were obtained invasively with a double luminal Swan Ganz catheter at the respective individual training loads. Training workloads were not increased further for the remainder of the training period. We found that none of the relevant cardiovascular variables measured (oxygen consumption, heart rate, mean arterial pressure, pulmonary arterial, and wedge pressure or peripheral systemic resistance) differed between the groups despite the fact that patients on the eccentric protocol worked at 397 W and the patients on the concentric protocol at only 97 W. These finding dispelled the initial fears of the cardiologists that mean arterial pressure, vascular resistance or pulmonary capillary pressure would be increased during eccentric exercise at relative high mechanical loads.

Steiner et al. (2004) analyzed a subset of the patients (*n* = 6 for eccentric and *n* = 6 for concentric training) studied by Meyer et al. (2003) with additional functional measurements, dual photon absorbtiometry (DXA) and muscle biopsies. He showed that patients trained eccentrically had a significant gain in muscle torque of 6–15%, depending on angular velocity. DXA, showed a small 3% gain in leg muscle mass both for the concentric and eccentric training regime. Additionally DXA showed leg and whole body fat mass to be decreased significantly in eccentrically trained patients. Muscle fiber size, capillarity and muscle ultrastructural composition remained unchanged with the exception of a non-significant 20% decrease in volume density of mitochondria in the group that performed eccentric training.

A number of additional studies have been published using eccentric training protocols in cardiac patients. Theodorou et al. (2013) performed a study with two groups of patients (*n* = 6 in each group) with heart failure that performed either stair ascending or descending for 6 weeks, three times per week. Gremeaux et al. (2010) evaluated the use of an eccentric ergometer vs. a standard ergometer in 14 patients with stable coronary disease. He exercised patients three times per week for 5 weeks either with a standard rehabilitation program or with an eccentric ergometer. All measured functional parameters improved similarly in both groups (200 m fast walk test, VO2peak, peak workload, 6 min walk distance and leg extensor strength). Only ankle plantar flexor strength increased significantly more with eccentric training (+17% vs. +7%). In a study of the same laboratory 30 patients with chronic heart failure (ejection fraction <45%; New York Heart Association class 2 or superior) were randomized to a concentric and an eccentric exercise group (Besson et al., 2013). Training was done for 7 weeks, three times per week for 30 min. Concentric exercise was done on a conventional ergometer at a workload corresponding to the first respiratory threshold (RPE 12-14). Eccentric exercise was done on an eccentric ergometer at 15 RPM at an RPE of 9-11. A more recent study was tailored to compare concentric to eccentric exercise training at a low rate of perceived exertion in patients with chronic heart failure (Casillas et al., 2016). All studies discussed above found eccentric exercise to be a safe training modality for patients with various cardiac conditions. The improvements seen with eccentric exercise protocols were similar to those obtained with conventional concentric training modalities but could be attained at a lower metabolic load and in some cases with additional benefits such as an increased muscle performance or volume.

## Moderate load eccentric exercise in sarcopenia

Many medical conditions list muscle wastage as a common symptom and as an indicator of poor prognosis. Among those, cardiac failure, COPD, chronic renal failure, chronic liver failure, and many malignant tumors come to mind. Sarcopenia (literally loss of flesh; Rosenberg, 1997), is also a natural process associated with aging accelerated in subjects older than 60 years. It is thus generally suggested that it is necessary to have patients and elderly subjects back to exercise to keep sarcopenia at bay and maintain quality of life and independence. Aging and the medical conditions mentioned above often go together with significant reductions in aerobic exercise capacity. This brings eccentric exercise protocols to the forefront as they can supply a potent muscle mechanical stimulus at a low metabolic cost. Moderate load eccentric exercise would therefore seem to be the exercise regime of choice in patient populations with limited exercise tolerance in general and in the elderly in particular.

It is recognized that with aging, changes in lifestyle such as the progressive decline of physical activity and poor nutrition are important contributors to sarcopenia. However, the compliance with programs aimed at modifying lifestyle has largely been unsuccessful. Based on these observations, it was suggested that hormone replacement therapy (HRT), could hold considerable promise both for women and men (Nedergaard et al., 2013b). HRT has fallen into disrepute in women because of adverse side effects, in particular an alleged increased incidence of ovarian cancer, associated with HRT (Beral et al., 2007). In men, loss of skeletal muscle mass is associated with the decline of testosterone production. Testosterone replacement therapy would therefore seem to be a logical choice. However, there are no large-scale prospective studies on the use of testosterone in eugonadal middle-aged and elderly men and there is a fear of an increase of cardiovascular risk factors as well as an increase of the risk for prostate cancer. Based on the evidence of estrogen and testosterone replacement therapy, (Nedergaard et al., 2013a) still propose hormone replacement therapy as a potentially viable way of reducing age-associated sarcopenia in men and also in women. In this context there might be a role in the future for selective estrogen and androgen receptor modulators (SERMS and SARMS, respectively; Nedergaard et al., 2013b) as they promise to deliver a more muscle tissue targeted drug action.

To take advantage of exercise regimes delivering high mechanical loads at low metabolic demands, a number of mostly explorative studies on the use of moderate load eccentric exercise have been carried out in the elderly population. An additional advantage of the use of eccentric exercise protocols in the elderly is seen in its capacity to elicit gains in muscle strength and volume at relatively low joint loads as discussed previously (LaStayo et al., 2014). Moreover, it may be promising in the future to combine HRT with eccentric load training protocols. It has indeed be shown that maximal eccentric exercise combined with HRT leads to a greater myogenic response in postmenopausal women than eccentric exercise alone (Dieli-Conwright et al., 2009, 2012).

Hortobágyi and DeVita (2000) subjected healthy elderly women (mean age 71 years) to standard concentric strength training or eccentric overload training for seven consecutive days. The eccentric overload group's combined strength gains were 1.8 times larger than those of the standard group. Perceived exertion and cardiovascular stress in terms of heart rate and mean arterial pressure was lower in the eccentric overload group. LaStayo et al. (2007) explored the safety and efficacy of an 11 weeks progressive eccentric resistance training program in elderly subjects. The capacity to perform negative work was increased 3-fold over this period and the gain in performance was accompanied by a 6% increase in thigh muscle mass (estimated by DXA). While markers of muscle damage and inflammation remained unchanged, there was a trend for insulin like growth factor to increase with eccentric training.

We compared conventional resistance exercise training (RT, *n* = 23) to moderate load eccentric exercise on an eccentric ergometer (ET, *n* = 23), in a study including 62 elderly men and women of average age 81 years (Mueller et al., 2009). Moderate load eccentric exercise training was carried out for 12 weeks with two sessions per week Maximal isometric leg extension strength increased significantly by 8.4% for the eccentric group only. Thigh lean mass was increased by some 2% in the eccentric and the concentric training group. We also found a significant loss of whole body (−5%) and thigh (−7%) fat tissue in the eccentric training group only. Muscle biopsies were taken from the vastus lateralis in 13 subjects of the concentric and in 14 subjects of the eccentric training group (Mueller et al., 2011). Muscle biopsies were analyzed by ultrastructural morphometry as well as by transcriptomic techniques. The gene expression profile of transcripts relevant for muscle growth, remodeling and repair as well as for transcripts relevant for muscle metabolism and mitochondria was established. An increase in IGF-1 was found to be associated with the muscle growth observed with both training modalities. The expression of transcripts encoding muscle growth, remodeling and repair were more increased in eccentric than in concentric exercise. Surprisingly, eccentric exercise decreased expression of genes encoding mitochondrial and metabolic transcripts. These changes in transcript levels were paralleled by significantly decrease in the volume fraction of mitochondria. A similar (but not significant) decrease in mitochondrial content was reported previously in the study of Steiner et al. (2004).

Eccentric exercise is seen as an option to prevent and reduce sarcopenia (Vásquez-Morales et al., 2013). The positive changes in muscle growth and repair combined with possible decreases in muscle mitochondria and metabolism call for further scrutiny and more work on eccentric exercise training protocols in particular when used to combat sarcopenia in the elderly.

## Moderate load eccentric exercise after ACL (anterior cruciate ligament) repair

Eccentric training protocols of many guises have become very popular in injury prevention as well as in rehabilitation of acute and chronic problems of the locomotor apparatus. Many of the published studies focus on eccentric exercise as a means for strengthening muscles or tendons. A general problem when looking at the literature consists in the bewildering number of options that exercise intervention in rehabilitative setting offers. We currently find many rather small studies with large multicenter studies with prospective randomization of patients and a-priori defined treatment protocols essentially missing. Despite the lack of rigor in many studies, eccentric exercise has become a solid part of the rehabilitation for many affections of the locomotor apparatus even though there is no definitive proof for the effectiveness of eccentric protocols. There is currently no generally agreed therapeutic best practices for the use of eccentric exercise in rehabilitative procedures of locomotor injuries or overuse (Hoppeler, 2014). In these settings moderate load eccentric exercise has been used specifically in the rehabilitation after ACL repair.

ACL injuries that need repair and subsequent rehabilitation are frequent in team sports such as soccer, basketball, handball etc. An ACL rupture rate of 8.6 per 10,000 game exposures has been reported by Dragoo (Dragoo et al., 2012). ACL ruptures are the most prevalent injury needing surgical treatment in recreational and competitive skiing (Sacco et al., 1998; Pujol et al., 2007). In skiing ACL ruptures are due to technical skiing errors with the added mechanical lever of the skis exposing the knee joint to disastrous loads. ACL ruptures in skiing are typically associated with meniscal tears and damage to other ligaments and thus pose significant rehabilitative problems.

Gerber et al. (2006) used moderate load eccentric exercise after ACL reconstruction in a case study. Negative work was applied progressively over a time period of 12 weeks starting 3 weeks after the surgical intervention. In a follow-up study Gerber et al. (2007a) randomly assigned 32 patients to either a 12-week traditional or eccentric exercise program. He could demonstrate that moderate load eccentric exercise could safely be implemented after ACL-reconstruction. He noted the absence of knee pain or effusions at any time of the training intervention. He further demonstrated the effectiveness of the training protocol with regard to quadriceps strength and hopping distance. These functional estimates of rehabilitative success improved significantly more in the group that used moderate load eccentric exercise than in the group receiving traditional rehabilitation.

A more systematic study involving 40 patients compared established rehabilitation after ACL-surgery to a rehabilitation program enhanced by progressive moderate load eccentric exercise (Gerber et al., 2007b). Patients used a self-selected cadence of 20–40 RPM and initial training durations of 5 min rated as “very light” on a Borg scale. Provided the knee remained free of pain and effusions, training time was increased up to 30 min and perceived exertion to “hard” in week 9–12. This study further compared surgical procedures namely bone –tendon- bone graft to semitendinosus—gracilis graft. Each subgroup comprised 10 subjects. Estimated by MRI, muscle volume of quadriceps and gluteus maximus increased more than double in the patients that trained eccentrically. Gains in muscle volume were not only found for the involved but also for the non-involved knee. A follow-up study conducted 1 year later re-assessed 17 patients in the eccentric and 15 patients in the conventional rehabilitation group (Gerber et al., 2009). Using MRI and functional tests the eccentric group still showed significantly larger quadriceps and gluteus maximus volumes. Likewise, quadriceps strength and hopping distance 1-year post surgery were still significantly better in the training group that received rehabilitation enhanced by moderate load eccentric exercise.

The studies above indicate that moderate load eccentric exercise is a potentially valuable tool in rehabilitation procedures of locomotor problems for which the emphasis is on gain of muscle volume and strength at low joint loads. In view of the better response of the quadriceps than the hamstring muscles noted in the ACL-rehabilitation studies above, it might be wise to complement moderate load eccentric exercise by training modalities aimed specifically at the hamstrings, such as the Nordic hamstring lowers (Hoppeler, 2014).

## Moderate load eccentric exercise in alpine skiing

Alpine skiing can arguably be characterized as the only sport in which well-coordinated eccentric muscle action is the decisive element. Eccentric muscle contractions are needed to dissipate the difference in potential energy between the start and the end of a ski run. We see a particularly important role of eccentric muscle action in alpine skiing competitions. This because, of the high speeds involved and the tight turns made possible by modern parabolic (carving) skis. Using accelerometers fixed to the sternum of athletes during world cup competitions we measured peak accelerations in excess of 3 g during turns. The centrifugal forces in a carved turn must be taken-up essentially only by the outer leg. This means that in 80 kg athletes, leg extensors must be capable of eccentrically resisting forces up to 3000 N (Spitzenpfeil, 2001). Due to the combination of high speed and small turning radius, g-forces were found to be largest in giant slalom.

**Figure 3:**
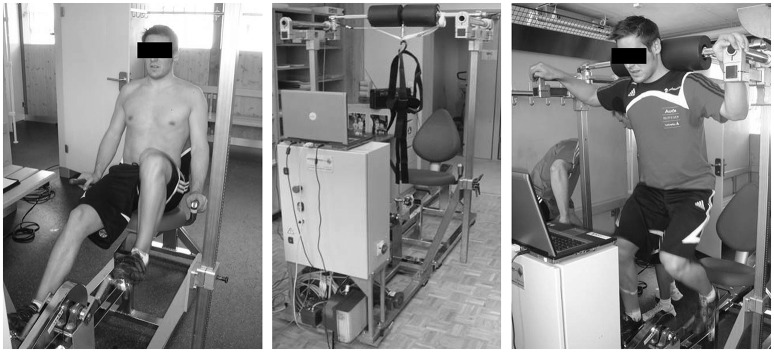
**Eccentric ergometer custom built for the Swiss National ski-team, capable of providing loads up to 2000 W**. As shown, this ergometer can be used in a sitting and in a standing position (with permission from Hoppeler, 2014).

As g-forces increase with increasing speed, coordinated eccentric leg strength is of paramount importance in elite competitive skiers. Furthermore, the very high eccentric loads are sustained under highly unstable dynamic conditions and for a prolonged period of time. The loading phase of muscles in skiing lasts several hundred milliseconds unlike the much shorter eccentric muscle activations in running or jumping. For a counter movement jump, muscle activation precedes joint movement by about 100 ms. In ski turns the EMG signal is closely synchronized to the eccentric muscle action and reaches maximal values toward the end of the eccentric movement (Berg and Eiken, 1999). The EMG signal during the eccentric phase of a turn is thus sustained for a much longer time period than in a jumping task. These differences in muscle activation patterns need to be considered in the implementation of effective strength training programs for alpine skiers. By choosing appropriate loads and cadences we have specifically tailored moderate load eccentric training programs to approximate the load characteristics experienced in alpine skiing. Moderate load eccentric training programs have thus been integrated into the preparation of the Swiss national ski team. For this purpose we built a motor driven eccentric ergometer capable of providing loads of up to 2000 W (Figure 3). This device allows for training leg muscles in a siting and in a standing position. As indicated above, in rehabilitative settings we have used similar devices training untrained subjects (and patients) progressively with loads up to 500 W. Elite skiers were trained with eccentric loads of up to 1200 W. Ski athletes could train at these loads after a small number of incremental training sessions without experiencing DOMS. 1200 W of eccentric load at a cadence of 60 RPM requires a torque development of approx. 2000 N per leg and per pedal cycle. As we trained athletes in four bouts of 5 min, they eccentrically resisted a total load of some 240 tons in a single training session. This is an order of magnitude more than what athletes would have experienced during conventional weight training sessions. This is also an indication of the extent of overload that can be reached with moderate load eccentric exercise in rehabilitative settings (Vogt and Hoppeler, 2011).

## Other uses of moderate load eccentric exercise

Moderate load eccentric exercise has been used in preventive or rehabilitative settings of various other medical conditions for which a gain in muscle mass at low metabolic cost or with low joint loads appeared advantageous. A short review of the still limited number of studies is given by LaStayo et al. (2014). He also provides details of the ramping protocols for moderate load eccentric exercise that his research group has given the acronym RENEW (for Resistance Exercise via Negative Eccentric Work).

### COPD (chronic obstructive pulmonary disease)

COPD patients exhibit severe muscle wasting due to a number of conditions. They suffer from general deconditioning, as well as systemic inflammation, poor nutrition, corticosteroid use and hypoxemia. The severe skeletal muscle dysfunction contributes importantly to the morbidity and mortality of this disease. In COPD, dyspnoe experienced during concentric exercise limits exercise tolerance. This makes COPD patients an obvious choice for moderate load eccentric exercise. Rooyackers et al. (1997) first explored eccentric exercise in COPD patients. He could demonstrate that eccentric exercise reduces ventilation by 35% at 50% of the maximal work capacity his patients were able to sustain. Rocha Vieira et al. (2011) used 5 weeks of progressive moderate eccentric exercise training up to a workload representing 60% of the peak oxygen consumption reached concentrically. His progressive protocol allowed patients to work with minimal muscle soreness, dyspnea, and muscle fatigue. At the end of the training period patients could work eccentrically at five time higher load for the same metabolic load and ventilator effort. Based on these results the authors support the use of eccentric training modalities in COPD patients.

### Cancer

Moderate load eccentric exercise has been used in a feasibility study including 20 older cancer survivors (50 breast, 25 prostate, 20 colorectal, and 5% lymphoma; LaStayo et al., 2010) with the complaint of moderate muscle weakness and fatigue. After 12 weeks of progressive load training (3 times per week) peak knee extension torque increased significantly by 11% and the timed up and go test (TUG) improved by 14%. Using a similar (12 weeks) progressive moderate load eccentric training program on ten men with prostate cancer, significant increases in knee extensor strength and quadriceps muscle volume were observed, independent of whether patients received androgen deprivation therapy (Hansen et al., 2009). Both studies indicate that eccentric exercise is well tolerated in cancer survivors and that patients can derive benefits in muscle strength and mobility from eccentric exercise protocols.

### Metabolic conditions

Muscle tissue is the main consumer of glucose. Exercise is thus of paramount importance in metabolically impaired subjects. In a population based study including 485 older adults with type 2 diabetes and 2133 subjects without, diabetes was associated with lower muscle strength and quality (Park et al., 2006). Exercise protocols that aim at improving muscle strength and volume are therefore recommended for this population (Sigal et al., 2006). Moderate load eccentric exercise programs have been used in patients with type 2 diabetes (Marcus et al., 2008, 2009). Marcus et al. (2008) compared aerobic exercise alone to aerobic exercise combined with moderate load eccentric exercise for 16-week in 15 diabetes type 2 patients. All patients improved glycemic control and physical performance. Additionally the group that used adjunct eccentric exercise showed additional improvements in thigh lean tissue and BMI (body mass index). In a study of 16 menopausal women with impaired glucose tolerance 10 subjects participated in a 12-week moderate load eccentric exercise training while six served as non-exercised controls. Eccentric exercise improved muscle mass and function without affecting insulin sensitivity negatively. From these preliminary studies it appears that moderate load eccentric exercise can safely be used in diabetes 2 patients. It should be noted however, that due to its inherently low metabolic cost, eccentric exercise may need to be complemented with conventional concentric exercise to maximize energy use and thus glucose disposal.

### Neurologic conditions

Progressive eccentric exercise training programs with small numbers of participants have been used for various neurological disorders. A group of 14 children with *cerebral palsy* (CP) were subjected to a progressive eccentric exercise protocol involving the elbow flexors (Reid et al., 2010). They were compared to an age matched group of normal developing children. With eccentric strength training CP children improved torque throughout the range of motion indicating decreased co-contractions. Twenty patients with *Parkinson's disease* were randomized to a 12-weeks progressive moderate load eccentric training program or to an active control group (Dibble et al., 2009). Muscle force, bradykinesia and the quality of life score (QOL) were improved more in the group that performed eccentric exercise. Nineteen patients with *multiple sclerosis* were randomized to either a standard exercise group or to a standard exercise and progressive moderate eccentric exercise group for 12-weeks (Hayes et al., 2011). In this setting, addition of eccentric training to a standard exercise intervention did not result in greater leg strength gains. Moreover, standard exercise alone improved balance and the ability to ascend or descend stairs more than the combined training modality. The equivocal results of eccentric exercise protocols in neurologic conditions call for more studies and caution in experimental design.

## Conclusions and outlook

Moderate load eccentric exercise or RENEW has been used effectively as a rehabilitative tool for medical conditions in which central energy supply is of concern. Its effectiveness in achieving muscle strength and volume gains at low metabolic loads has amply been demonstrated. Moderate load eccentric exercise can lead to similar improvements of muscle performance than conventional strength training protocols but at lower joint loads. This makes it a valuable tool in orthopedic rehabilitation and injury prevention. There is a distinct lack of large-scale multi-center studies prospectively exploring eccentric exercise modalities making the currently available evidence essentially based on exploratory-type studies. We also need to acknowledge that the basic physiological mechanisms by which muscle produce force when lengthened are still debated (Hoppeler, 2014; Hoppeler and Herzog, 2014; Herzog et al., 2016).

## Author contributions

The author confirms being the sole contributor of this work and approved it for publication.

### Conflict of interest statement

The author declares that the research was conducted in the absence of any commercial or financial relationships that could be construed as a potential conflict of interest.
